# New insights into the molecular mechanism of rhodopsin retinitis pigmentosa from the biochemical and functional characterization of G90V, Y102H and I307N mutations

**DOI:** 10.1007/s00018-021-04086-0

**Published:** 2022-01-07

**Authors:** María Guadalupe Herrera-Hernández, Neda Razzaghi, Pol Fernandez-Gonzalez, Laia Bosch-Presegué, Guillem Vila-Julià, Juan Jesús Pérez, Pere Garriga

**Affiliations:** 1 Departament d’Enginyeria Química, Grup de Biotecnologia Molecular i Industrial, Centre de Biotecnologia Molecular, Universitat Politècnica de Catalunya, Edifici Gaia, Rambla Sant Nebridi 22, 08222 Terrassa, Catalonia Spain; 2 The Tissue Repair and Regeneration Laboratory (TR2Lab), University of Vic-Central University of Catalonia, Sagrada Família, 7, 08500 Vic, Catalonia Spain; 3 Present Address: Campo Experimental Bajío, INIFAP, Km 6.5 Carretera Celaya-San Miguel de Allende, CP 38110 Celaya Gto, Mexico

**Keywords:** Retinal degenerative diseases, G protein-coupled receptors, Protein folding, Conformational stability, Ligand binding

## Abstract

Mutations in the photoreceptor protein rhodopsin are known as one of the leading causes of retinal degeneration in humans. Two rhodopsin mutations, Y102H and I307N, obtained in chemically mutagenized mice, are currently the subject of increased interest as relevant models for studying the process of retinal degeneration in humans. Here, we report on the biochemical and functional characterization of the structural and functional alterations of these two rhodopsin mutants and we compare them with the G90V mutant previously analyzed, as a basis for a better understanding of in vivo studies. This mechanistic knowledge is fundamental to use it for developing novel therapeutic approaches for the treatment of inherited retinal degeneration in retinitis pigmentosa. We find that Y102H and I307N mutations affect the inactive–active equilibrium of the receptor. In this regard, the mutations reduce the stability of the inactive conformation but increase the stability of the active conformation. Furthermore, the initial rate of the functional activation of transducin, by the I307N mutant is reduced, but its kinetic profile shows an unusual increase with time suggesting a profound effect on the signal transduction process. This latter effect can be associated with a change in the flexibility of helix 7 and an indirect effect of the mutation on helix 8 and the C-terminal tail of rhodopsin, whose potential role in the functional activation of the receptor has been usually underestimated. In the case of the Y102H mutant, the observed changes can be associated with conformational alterations affecting the folding of the rhodopsin intradiscal domain, and its presumed involvement in the retinal binding process by the receptor.

**Supplementary Information:**

The online version contains supplementary material available at 10.1007/s00018-021-04086-0.

## Introduction

Rhodopsin (Rho) is the photoreceptor protein found in the disk membranes of the rod outer segment (ROS) of retinal rod photoreceptor cells and responsible for scotopic vision at low light intensities [1]. More specifically, Rho is a class A G protein-coupled receptor (GPCR) and it is widely known for being the first GPCR whose crystallographic structure was solved at atomic resolution [2]. Rho has been widely used as a suitable model for structural studies of other GPCRs [3]. From the physiological side, mutations in GPCRs are associated with different pathologic conditions and are responsible for causing a wide range of human diseases. In particular, naturally occurring mutations in the visual GPCR Rho cause inherited retinal degenerative diseases, such as retinitis pigmentosa (RP) [4]. Structural aspects of this photoreceptor protein have been intimately linked to genetic retinal neurodegenerative diseases [5] and unravelling the molecular and cellular consequences of RP mutations is paving the way for the development of potential novel therapies [6].

Rho mutations associated with RP have been extensively characterized by means of heterologous expression of the mutated proteins in mammalian cell systems. Bovine Rho has been widely used to conduct systematic structure–function studies of Rho mutations. Many of these amino acid substitutions have been characterized to elucidate the molecular mechanisms that accompany retinal degenerative diseases, such as RP. The best cell platforms for obtaining recombinant Rho have been HEK293 [7] and COS-1 [8] mammalian cell systems.

The Rho mutant G90V was first identified in a Swiss family of three generations, which showed a typical phenotype of an autosomal dominant form of RP, with marked fundus changes developing in later stages of life [9]. Subsequent to this discovery, studies regarding the G90V mutant have used heterologous expression systems and immunopurification strategies to deepen the knowledge on the structural details underlying the molecular mechanisms of the disease [10, 11]. Furthermore, the deleterious effect of the G90V mutation could be partially reversed in vitro by means of 9-cis-retinal alone [11] or in combination with other ligands like the flavonoid quercetin [12].

The Y102H and I307N mutants were developed in chemically mutagenized mice to study and characterize the disease [13]. The interest in these two mutations comes from the fact that there is no need to overexpress the protein, because the mutation is already integrated into the mouse genome, thus avoiding concerns associated with overexpression that can cause retinal degeneration per se. Although these mutations have not been reported in humans so far, they are very useful as models for deciphering the molecular details of the retinal degenerative process associated with RP. These models can prove extremely helpful for advancing clinical studies and for future therapeutic developments. In fact, it was found that these mutations showed phenotypic similarity to human B1 type Rho mutations. Patients with class B mutations display a slower disease progression, and may be subdivided into class B1 and B2 [14]. Rod degeneration is focal in class B1 and these mutations exhibit impaired deactivation of phototransduction after exposure to high intensity light flashes [15].

In our current study, we find that the Y102H and I307N mutations shift the inactive–active equilibrium of the receptors towards the active conformation. The I307N mutant shows an altered transducin activation which may be associated with structural changes from the top of helix 7 to the C-terminus of Rho. In the case of the Y102H mutant, this mutation may affect Rho folding (and retinal binding) at the intradiscal domain of the receptor. Our results provide new insights into the mechanisms underlying RP induced by Rho specific mutations and can help in designing novel therapeutic strategies for the treatment of RP.

## Materials and methods

### Materials

11-*cis*-retinal (11CR) was kindly provided by the National Eye Institute, National Institutes of Health (Bethesda, MD, USA), mAb rho-1D4 antibody was purchased from Cell Essentials (Boston, USA) and was coupled to Cyanogen bromide (CNBr)-activated Sepharose 4B beads. *n*-dodecyl-β-d-maltoside or dodecyl maltoside from Anatrace Inc. (Maumee, OH, USA), Nonamer–peptide H-TETSQVAPA-OH was synthetized by Unitat de Tècniques Separtatives i Sìntesi de Pèptids (Barcelona, Spain), Polyethyleneimine (PEI) was provided by Polysciences Inc. (Warrington, PA, USA), Cellulose membrane and manifold for radioactivity assay was purchased from Millipore, (France) and the [S^35^]GTPγS was purchased from Merck. All other chemicals were purchased from either Fisher (Darmstadt, Germany) or Sigma (Madrid, Spain).

Cell culture materials. COS-1 cells were obtained from the American Type Culture Collection (Manassas, Virginia), HEK 293S GnTI¯ cells (used for the production of WT and Rho mutants for electrophoretic and immunofluorescence assays) were from ECAC (Salisbury, UK), culture media, Dulbecco’s modified Eagle medium (DMEM)), fetal bovine serum, L-glutamine and penicillin–streptomycin were obtained from Sigma-Aldrich (Madrid, Spain) and DMEM-F12 was purchased from Lab Clinics (Barcelona, Spain). Opti-MEM reduced serum media was from Invitrogen (Barcelona, Spain).

#### Buffers

PBS1X or buffer A (137 mM NaCl, 2.7 mM KCl, 1.5 mM KH_2_ PO_4_, and 8 mM Na_2_HPO_4_, pH 7.4), elution buffer 1 or buffer B (0.05% dodecyl maltoside in buffer A, pH 7.4), elution buffer 2 or buffer C (0.05% dodecyl maltoside in buffer A, pH 6), transducin (Gt) buffer (25 mM Tris, pH 7.5, 100 mM NaCl and 5 mM magnesium acetate), TBS (137 mM NaCl and 10 mM Tris pH 8), TTBS (0.1% Tween 20 in TBS).

### Methods

#### Construction of opsin mutants

G90V mutant was already available from previous studies [11]. The other two mutations, Y102H and I307N, were newly designed by means of a site-directed mutagenesis kit (QuikChange, Stratagene) on a synthetic bovine opsin gene [8] using the pMT4 vector as a template. The following primers were used: forward primer Y102H (mutation TAC → CAC): CCTCTCTCCATGGGCACTTCGTCTTTGGG; reverse primer Y102H: CCCAAAGACGAAGTGCCCATGGAGAGAGG; forward primer I307N (mutation ATC → AAC): CCCGGTCATCTACAACATGATGAACAAGCAGTTCC; reverse primer: GGAACTGCTTGTTCATCATGTTGTAGATGACCGGG. Primers were synthetized by Sigma. The mutant plasmids were sequenced to verify the correct introduction of the mutations.

#### Expression and purification of bovine recombinant wild type (WT) Rho and mutants

The expression and purification of the visual receptors were performed as described previously [12]. Plasmids encoding the WT and mutant Rho genes were expressed in transiently transfected COS-1 cells plates at 85% confluence by chemical transfection using PEI (100 µL at 1 mg/ml) with 30 µg of plasmid DNA/145 mm plate. Cells were harvested 48 h after transfection and washed three times with buffer A. Opsins were reconstituted with 15 µM of 11CR in buffer A by overnight incubation at 4 °C. Subsequently, cells were solubilized with dodecyl maltoside (1% w/v), 100 µM of phenylmethanesulfonyl fluoride (PMSF) and protease inhibitor cocktail by shaking 1 h at 4 °C, finally, they were ultracentrifuged for 30 min at 35000 rpm. The pigments from the supernatant were purified by immunoaffinity chromatography using CNBr-activated sepharose coupled to rho-1D4 antibody. The resin was washed with buffer B and the bound pigments were eluted with buffer B containing 100 µM 9-mer peptide. All procedures, up to and including binding of the receptor to the immunoaffinity matrix were performed at 4 °C in the dark or under dim-red light.

#### Subcellular localization of mutant opsins

Cellular opsins localization was conducted by means of immunofluorescence microscopy as previously described [16]. Briefly, a low density of HEK-293S GnTi¯ cells was seeded in six-well plates containing sterile coverslips and incubated for 24 h at 37ºC, 5% CO_2_. Next day, the old medium was removed and the cells were transfected as described previously. 24 h after transfection, the solution was removed and the cells were washed twice with 3 mL PBS (buffer A) and immediately fixed with 1 mL of a mixture containing 37% formaldehyde and 15% methanol in water at 37 °C for 20 min and consecutively cells were washed with buffer. Sample was blocked with 5% skim milk in TBS stirring for 30 min. Cells were washed again three times by shaking with 2 mL of TTBS for 10 min. Rho-1D4 antibody (dilution 1: 2000 in TBS) was added and the cells were incubated for 1 h by shaking and washed three times (2 mL TTBS buffer for 10 min). Subsequently, the goat anti-mouse secondary antibody tagged with FITC (1:200 dilution in TBS) was added and cells were incubated for 1 h and washed as described. Coverslips containing the cells were mounted on a glass slide with the help of Vectashield Mounting Medium containing DAPI (Vector Labs, RU). Images were collected using a fluorescence microscopy system, Nikon eclipse Ti equipped with a DS-QiMc camera.

#### Western blot analysis

Electrophoresis analysis was carried out to compare the patterns of the purified mutant opsins with that WT receptor. For Western blot analysis, the monoclonal Rho-1D4 antibody was used as the primary antibody and anti-mouse as secondary antibody.

#### UV–visible spectral characterization

For spectroscopic characterization of Rho samples, a Varian Cary 100 Bio spectrophotometer (Varian, Australia) was used. Temperature was controlled by a Peltier accessory equipped with a water-jacketed cuvette holder connected to a circulating water bath. All spectra were recorded in the 250 nm–650 nm range with a bandwidth of 2 nm, a response time of 0.5 s and a scan speed of 400 nm/min.

#### Photobleaching and acidification of purified Rho

Samples were illuminated with a 150-W Dolan–Jenner MI-150 power source, equipped with an optic fiber guide and using a 495 nm cutoff filter for 90 s to ensure complete photoconversion to the 380 nm absorbing species. Acidification was carried out immediately after photobleaching by the addition of 2 N H_2_S0_4_ which yields a pH ~ 2.0 and the absorption spectrum was subsequently recorded. The re-protonation of the Schiff base nitrogen caused by acidification shifts the *A*_λmax_ to 440 nm.

#### Hydroxylamine reactivity and thermal stability

A solution of 1 M hydroxylamine hydrochloride (adjusted to pH 7) was added to dark-adapted samples in a spectroscopic cuvette (final concentration of 50 mM), and successive spectra were recorded every 5 min to monitor the decrease of *A*_λmax_ and formation of retinaloxime. The reactions were carried out in the dark at 20 °C. The initial rate was obtained by linear regression fitting the experimental data, and for the G90V mutant the linear portion of the curve was used for better accuracy.

Thermal stability of Rho was studied by monitoring the decrease of *A*_max_ of the visible spectral band as a function of time at 48 °C. Spectra were recorded every 5 min and half-life times were determined by fitting the experimental data to single exponential curves.

#### Rho regeneration

For the regeneration experiments, 2.5-fold molar excess of 11CR (stock solution in ethanol) was added over dark adapted samples in the spectroscopic cuvette and thoroughly mixed. Immediately after, the sample was illuminated with a yellow cutoff filter (> 495 nm) to avoid photobleaching of the free retinal and successive spectra were registered every 10 min in the case of WT and every minute in the case of G90V mutant at 20 °C in the dark until no further increase in A_λmax_ was observed.

#### Metarhodopsin II (Meta II) decay

Meta II decay experiments were performed on a PTI QuantaMaster 4 spectrofluorometer equipped with a TLC50 cuvette holder Peltier accessory, for temperature control. Initially, the Trp fluorescence of a dark-adapted sample was recorded at 20 °C until a steady base line was obtained. After that, the sample was illuminated for 30 s with a 150-W Dolan–Jenner MI-150 power source using a cutoff filter (> 495 nm) and the fluorescence intensity was monitored until it reached a plateau. All fluorescence spectra were carried out by exciting the samples for 2 s at 295 nm, using a slit bandwidth of 0.5 nm, and blocking the excitation beam for 28 s with a beam shutter to avoid photobleaching of the sample. Trp emission was monitored at 330 nm with a slit bandwidth of 10 nm. The half-life time (*t*_1/2_) of the fluorescence increase was fit to a single exponential function.

#### Transducin activation assay

Transducin activation was monitored with a radionucleotide filter binding assay. In this assay, GTPγ^35^S uptake by purified transducin, from bovine retinas, upon binding to activated Rho, was measured. The assay was performed by mixing 10 nM of WT or mutant with 500 nM of transducin in Gt buffer containing 5% of glycerol, 2.5 mM dithiothreitol (DTT) and 5 µM of [S^35^]GTPγS (1250 Ci/mmol). The reaction was initiated by the addition of the sample in dark state and filtered after different incubation times (every 4 min), either in the dark (0, 4 and 8 min) or after illumination (12, 16, 20, 24 and 28 min) to determine the amount of bound [S^35^]GTPγS.

#### Molecular modeling analysis

##### System preparation

Active and inactive conformations of WT Rho were retrieved from the Protein Data Bank (PDB) with PDB accession codes 5DYS [17] and 1U19 [18], respectively. The 5DYS structure was edited in such a way that all residues corresponded to the WT Rho sequence. Parametrization of both 11CR and ATR bound to Lysine 296 was carried out following a procedure previously described [19]. Parameters for the lysine–retinal residue were obtained using the General Amber Force Field. [20]. Charges were generated with the restrained electrostatic potential at the HF/6-31G* level, the default charge generation approach applied in Amber force fields [21, 22].

To construct the structures of the Y102H and I307N mutants, residues 102 and 307 were replaced by the corresponding ones in the mutant forms His and Asn, respectively. Missing atoms from these residues were added using the *LEaP* module from Amber18 software [21].

Subsequently, each of the Rho structures were inserted in a membrane composed of POPC lipids parametrized with the Lipid17 force field of AMBER18 [21] and with a NaCl concentration of 0.15 nM, constructed using the *Packmol*–*Memgen* package [23].

##### Molecular dynamics of WT and mutants Y102H and I307N

Molecular Dynamics (MD) simulations were carried out to model the receptor structures in the same environment in vitro experiments are conducted. All calculations in the present work were performed with the particle mesh Ewald MD code of AMBER18 software in its CUDA version, using the AMBER ff14SB force field [20].

Previous to MD simulations, each system was first relaxed, heated up and density equilibrated, as previously described [19]. After this preliminary step, a 500 ns MD simulation was performed for each system including the WT in its active and inactive forms as well as the mutants, to ensure the stability of the corresponding systems. However, only the last 50 ns of each trajectory were considered for the subsequent analysis.

##### Free-Energy Decomposition Calculations

To determine the contribution of each residue to the internal energy of the structure we used the MMGBSA decomposition method, [23] by means of the *MMPBSA.py* module of AMBER18 [24]. Energy decomposition was performed on a pairwise per-residue basis, which means that the contribution of each residue with each residue of the receptor has been computed separately. All energy components were calculated using only the snapshots of the last 50 ns of each MD simulation from each complex.

##### Clusterization

Similar structures from each simulation were grouped in clusters using the average linkage algorithm, [25] implemented in the *cpptraj* module of AMBER18. This procedure was performed to identify the stereochemical features that characterize the different Rho complexes. For this purpose, the RMSD of Cα in the transmembrane (TM) helices was used as distance.

## Results

We have previously characterized the molecular features of the G90V Rho mutant [11]**.** The other two mutations, Y102H and I307N, were newly designed and obtained by means of site-directed mutagenesis and the correct introduction of the mutations was confirmed by sequencing the mutated genes. A preliminary version of some of the results herein discussed has been previously reported [26].

Rho synthesis and degradation are highly regulated processes and several mutations can affect them by causing misfolding and aggregation. Furthermore, some mutations can impair opsin transport to the ROS membrane. In this regard, the heterologous expression of rod WT and mutant opsins in cell culture can be used to study protein biogenesis, trafficking, aggregation and degradation [2].

As reported in previous studies, WT opsin traffic to the membrane is a very efficient process, and most of the synthesized proteins can be found in the membrane. We analyzed the location of the RP mutant opsins and we did not find large changes in the profile of the mutants except that in some cases they showed some apparent retention in the ER and also the formation of intracellular inclusions, especially for the G90V mutant (Fig. S1, supplementary material) in agreement with previous results [11].

The immunopurified WT Rho and mutants were characterized by Western blot using a sample of purified Rho from ROS as a control (Fig. S2, supplementary material). A characteristic smear typically observed in Western blots of Rho expressed in COS-1 cells and usually attributed to heterogeneous glycosylation, can be observed in all the samples, but it particularly appears to be more intense in the case of the G90V mutant (Fig. S2, lane 3, supplementary material). In all cases, the Rho monomeric band is clearly observed, but the corresponding dimer band can only be clearly detected in the case of Rho obtained from ROS, the control sample. In the case of the G90V mutant, however, the presence of the band corresponding to the dimer species is partially occluded by the intense smeary pattern observed for this protein. For Y102H and G90V, a clear band is observed below the Rho monomer band. The presence of a band around 27 kDa may correspond to a truncated protein form as previously described [10, 16, 27]. In the case of the I307N mutant, two minor bands can be detected below the 40 kDa main opsin band that could also correspond to a truncated protein.

### UV–Vis spectroscopic characterization

**Fig. 1:**
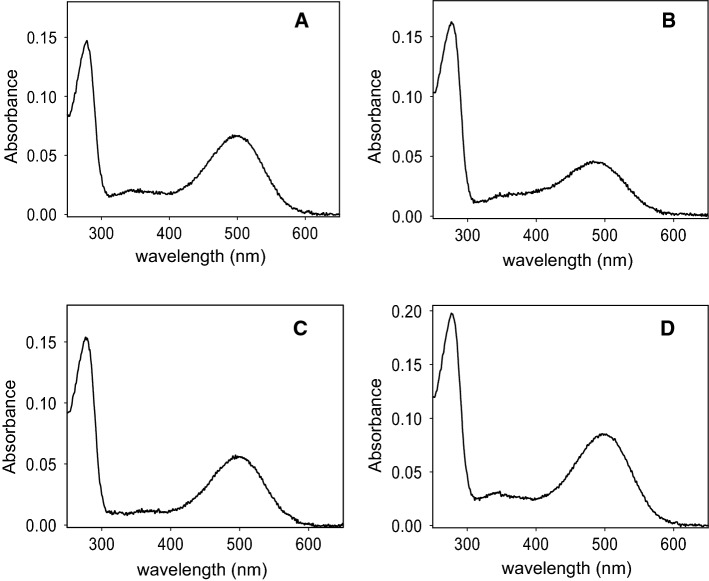
UV–visible absorption spectra of WT Rho and G90V, Y102H and I307N mutants in the dark state. Samples were in PBS pH 7.4 containing 0.05% dodecyl maltoside. Spectra were recorded at 20 °C. **A** WT, **B** G90V mutant,  **C** Y102H mutant, **D** I307N mutant

The purified WT and mutants were also analyzed by UV–Vis spectroscopy and the corresponding spectra were recorded under dark conditions at 20 °C (Fig. 1). The Y102H and I307N mutants showed a spectroscopic behavior similar to the WT in agreement with previous results [13], with a maximum absorption band in the visible region at 498 nm and 500 nm, respectively. In the case of the G90V mutant, a blue shift of 10 nm that had been previously reported, was observed [10, 11]. A summary of the spectral parameters, including the absorbance value of the visible chromophoric band, ε and the spectral *A*_280_/*A*_λmax_ ratio are shown in Table 1.

**Table 1:** Spectroscopic properties of WT and mutants

	WT	G90V	Y102H	I307N
λ_max_	500	490	498	500
Ratio (*A*_280_/*A*_λmax_)	2.30 ± 0.20	3.73 ± 0.23	2.70 ± 0.12	2.20 ± 0.04
ε × 10^3^ (M^−1^·cm^−1^)	42.2 ± 2.2	37.8 ± 0.9	37.6 ± 1.3	43.9 ± 0.4

The G90V and Y102H mutants showed a slight increase in the ratio, probably due to the fact that the introduction of this mutation causes a small fraction of misfolded protein and/or leads to decreased structural stability. These mutants also have a very similar molar extinction coefficient. On the other hand, the mutant I307N presents a ratio and ε more similar to that of WT Rho.

### Photobleaching and acidification of purified Rho

**Fig. 2:**
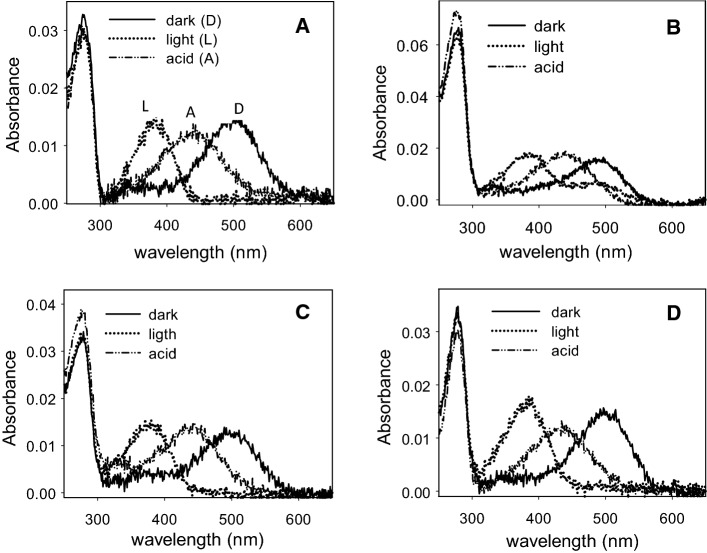
UV–visible characterization of the immunopurified WT and RP mutants. Dark state (solid line), photobleaching (dotted line) and acidification (dashed line). Samples in PBS pH 7.4 buffer containing 0.05% dodecyl maltoside. Spectra were recorded at 20 °C. **A** WT, **B** G90V mutant, **C** Y102H mutant, **D** I307N mutant

The UV–Vis spectra of WT and the mutants were recorded in the dark, upon illumination for 30 s and after subsequent acidification (Fig. 2). The main difference observed was in the G90V mutant which did not show a complete conversion of the visible band to 380 nm upon illumination. This remaining band (about 40% of the dark visible band) had a similar λmax as the dark pigment, suggesting a possible conversion to a photointermediate with a retinal binding pocket similar to the dark pigment, including the presence of a protonated Schiff base linkage [28]. Acidification of these photoactivated receptors resulted in a similar behavior, showing a band with a maximal absorbance at 440 nm corresponding to the protonated Schiff base.

### Hydroxylamine reactivity and thermal stability

**Fig. 3:**
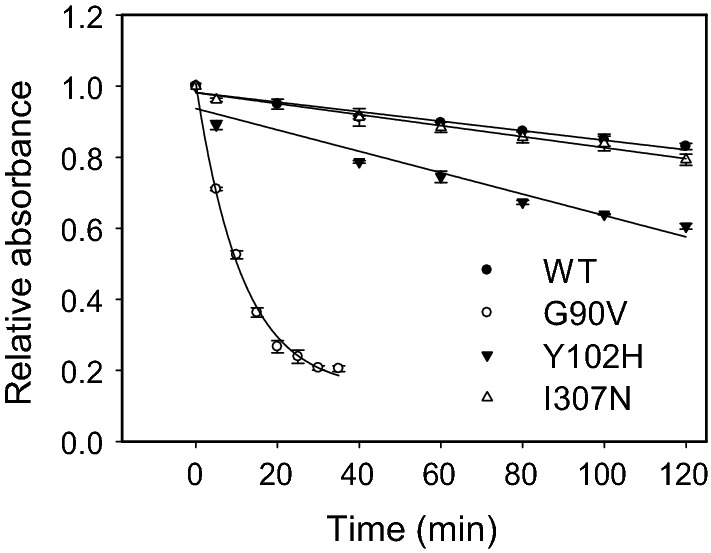
Chemical stability of WT, G90V, Y102H and I307N. Samples purified in PBS pH 7.4 and 0.05% dodecyl maltoside were incubated with 50 mM hydroxylamine pH 7 and the decrease of Abs at λ_max_ was recorded over time at 20 °C. **●** WT rhodopsin, ○ G90V, ▼ Y102H, and I307N. Mean value and standard error (SE) obtained in independent purifications (n = 3)

**Fig. 4:**
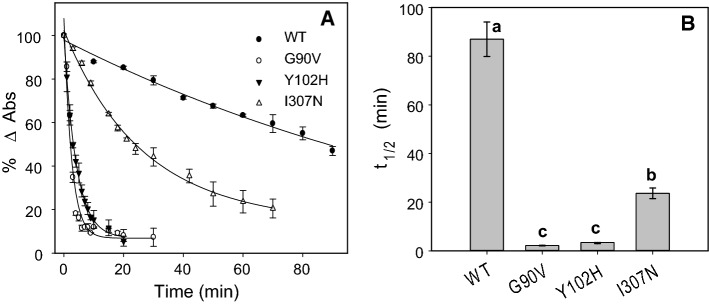
Thermal stability of WT and G90V, Y102H and I307N. Immunopurified WT and mutants in PBS pH 7.4 and 0.05% dodecyl maltoside were incubated at 48 °C. The normalized Abs values at *λ*_max_ were plotted as a function of incubation time (**A**) and the *t*_1/2_ was calculated (**B**). Mean values with different letters are significantly different at *P* < 0.05. Mean value and standard error (SE) obtained in independent purifications (n = 3)

Hydroxylamine is a compound that is used in Rho studies to determine whether a mutation can affect the structural compaction in the Schiff base environment. This reagent can enter the retinal binding site and break the Schiff base linkage when the protein has an open conformation [29]. The WT and RP mutants in the dark state were treated with hydroxylamine, which causes a decrease in the visible Amax with time (Fig. 3). As expected, the Schiff base in WT Rho was remarkably stable in the presence of hydroxylamine under dark conditions due to the compact structure around the Schiff base linkage. In contrast, the G90V mutant showed a dramatic decrease in the visible band due to the less compact structure in the Schiff base linkage environment [10, 11]. In addition, for the Y102H mutant, a faster decrease in Amax was observed but not as marked as in the case of G90V mutant. On the other hand, the I307N mutant showed a similar behavior when compared to the WT, indicating that this RP mutant has better structural compaction around the Schiff base. For a better comparison, the initial rate for the process was calculated and then normalized regarding to WT (taken as 1.0). It was also noticed that hydroxylamine reactivity was 38.5 times faster for G90V mutant when compared to the WT and 4.5 and 1.8 times faster for Y102H and I307N mutants, respectively (Table S1, supplementary material). As for the thermal stability assay monitored at 48 °C, the G90V and Y102H mutants were very unstable in the dark state (Fig. 4), showing very fast thermal bleaching kinetics (2 and 3 min, respectively). I307N receptor was also somewhat unstable, although to a lower extent, with a t_1/2_ of 23 min, four times faster than WT Rho.

### Chromophore regeneration

**Fig. 5:**
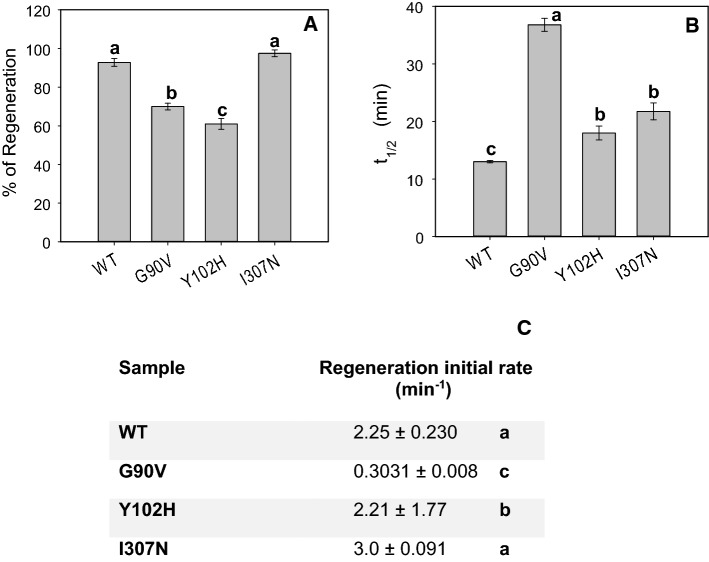
**A** Percentage of regeneration of photoactivated WT and RP mutants. 2.5 fold of 11CR was added to dark adapted immunopurified WT and mutants in PBS pH 7.4 containing 0.05% DM and the percentage of regeneration was determined after pigment illumination with cut off filter > 495 nm to avoid photobleaching of the free retinal. To determine the extent of chromophore regeneration successive spectra were recorded every 5 min at 20 °C until no further increase in A_max_ was detected. Mean values with different letters are significantly different at P < 0.05. **B** Meta II decay of WT and G90V, Y102H and I307N mutants. Mean values with different letters are significantly different at P < 0.05. Mean value and standard error (SE) obtained in independent purifications (n = 3). **C** Initial rates for the chromophore regeneration of WT and the RP mutants. Mean values in a column with different letters are significantly different at P < 0.05

In the experiment of pigment regeneration with 11CR after Rho photobleaching, the percentage (Fig. 5A), and the initial rate of regeneration (Fig. 5C) were analyzed. In this assay, Y102H and G90V mutants presented the lowest percentage of regeneration compared to the WT (61 and 70%, respectively). The I307N mutant showed a similar total regeneration as the WT Rho. This result agrees with the amount of regenerated protein obtained during the purification of these receptors, where the highest yield was for the I307N mutant and the WT, and the lower yield for the Y102H and G90V mutants (Fig. 1).

The G90V mutant was the one with the slowest regeneration rate (Fig. 5C). In the case of the Y102H mutant, despite having the lowest percentage of regeneration (Fig. 5A), it showed the same regeneration rate as the WT (Fig. 5C). On the other hand, the I307N had a slightly faster percentage of regeneration compared to WT Rho (Fig. 5C).

### Meta II decay

The stability of the active state of purified WT and mutants was carried out by means of fluorescence spectroscopy following the Trp fluorescence increase upon illumination. For all mutants, the Meta II stability reflected a slower decay when compared with the WT (13 ± 0.20 min) (Fig. 5B). The highest difference was reported for the G90V mutant, with a t_1/2_ of 36.0 ± 1.1 min. This result agrees with previous studies reported for this mutant [11].

In contrast, Y102H and I307N mutants had a t_1/2_ of 18.0 ± 1.2 min and 21.8 ± 1.5 min, respectively. A previous study carried out showed similar behavior for these mutants, where both presented higher MetaII decay values compared to the WT (WT = 5.9 min, Y102H = 7.5 min and I307N = 7.9 min), with a slightly longer time for I307N mutant [13]. The differences previously reported appear no to be statistically significant due to the large standard error in the experimental values [13]. The different values presented here, compared with those reported in that previous study, are likely due to the experimental conditions in which the experiment was carried out, since the buffer used was 2 mM sodium phosphate, pH 6.7 and the dodecyl maltoside concentration was 0.1%. In our experiment, the buffer was PBS pH 7.4 and the dodecyl maltoside concentration was 0.05%. In this regard, the critical effect of pH and dodecyl maltoside detergent concentration on the stability and function of Rho have been already reported [30, 31]. In addition, the actual Rho concentration used in this previous study is not specified (the protein absorbance values provided are normalized) [13] and this can also affect the experimental values obtained. It is generally accepted that all these experimental conditions can significantly affect the particular experimental values of the reported assays in Rho studies. Furthermore, the previously reported study, where the two mutations were described for the first time, was carried out using a mutant with an engineered disulfide bond [13] that is inherently more stable than ours.

### Gt activation of purified WT and RP Rho mutant

The function of Rho is to activate Gt and initiate the visual signal transduction cascade. Gt activation involves the exchange of guanosine diphosphate for guanosine triphosphate which results in the dissociation of the Gt α-subunit from the Gβγ heterodimer [32]. The ability of purified WT and RP mutants to catalyze guanine nucleotide exchange by Gt was measured using a radioactive filter-binding assay as described previously in the *Materials and Methods* section.

**Fig. 6:**
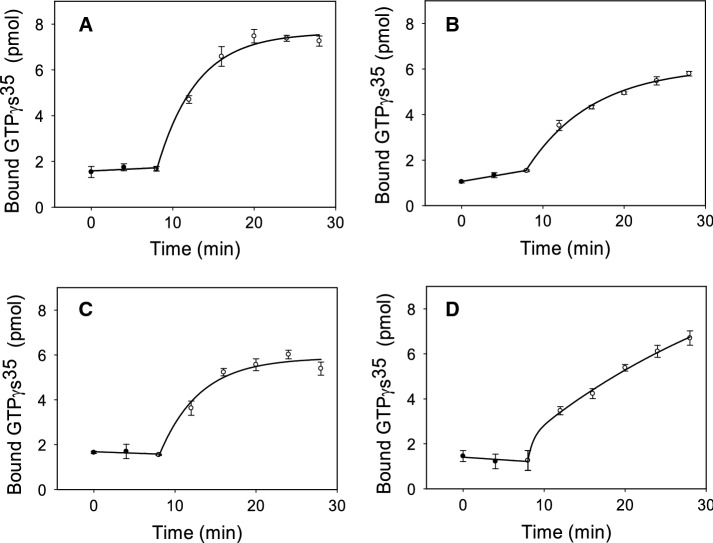
Gt activation by WT and G90V, Y102H and I307N mutants. Gt activity was measured by means of a radionucleotide filter-binding assay in transducin (Gt) buffer. The reaction was initiated by the addition of the WT or mutants, and samples were filtrated at different times in the dark and after illumination. **A** WT, **B** G90V mutant, **C** Y102H mutant, **D** I307N mutant. Mean value and standard error (SE) obtained in independent purifications (n = 3)

The photoactivated Y102H and G90V mutants bound and activated Gt with a similar kinetics to that of the WT (Fig. 6). However, the amount of GTPγ^35^S bound was somehow lower compared to the WT. In addition, the I307N mutant showed a significantly altered kinetics that kept increasing with time. In the case of the G90V mutant there was a detectable increase of the activation in the dark before illumination and the Y102H mutant showed a lower total activation level than the WT. The Gt initial activation rates obtained from the linear portion of the curves were obtained and analyzed for statistical significance and all the three mutants showed slower rates when compared to the WT receptor (Table 2).

**Table 2:** Gt initial activation rates were calculating from the linear portion of the curves in Fig. 6 and the statistical analysis was performed

Sample	Gt initial activation rate (min^−1^)
WT	0.9755 ± 0.0134^a^
G90V	0.5680 ± 0.0184^b^
Y102H	0.7055 ± 0.0714^ab^
I307N	0.5455 ± 0.1266^b^

Mean values in a column with different letters are significantly different at *P* < 0.05

### Molecular modeling analysis

MD simulations were performed to assess structural and stability differences that may be attributed to site mutations. For this purpose, 500 ns simulations were carried out of the WT and Y102H and I307N mutants in the inactive state together with a 500 ns simulation of the WT in its active state.

**Fig. 7:**
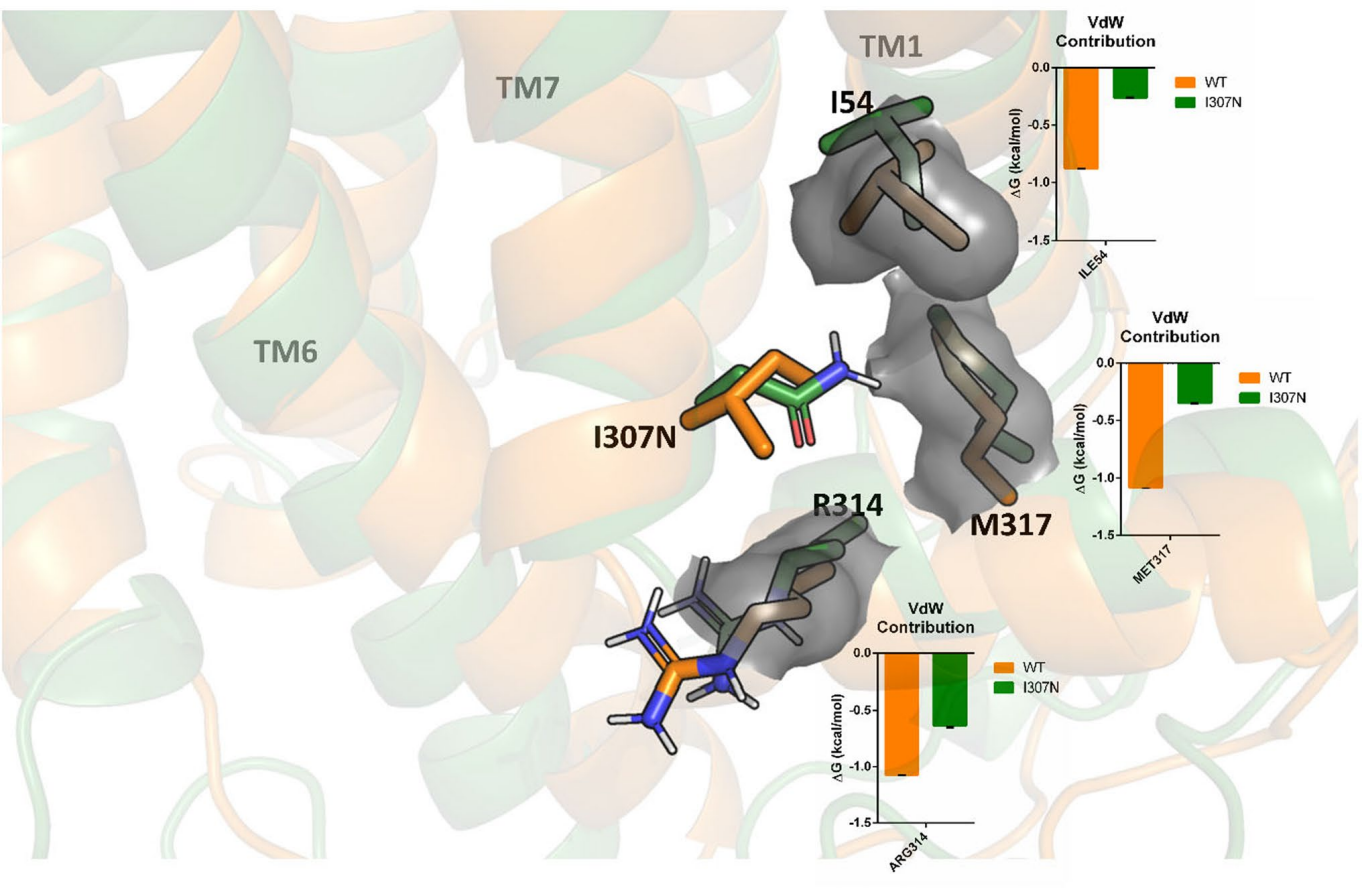
Comparison of WT and I307N mutant on their inactive states. WT (orange) and I307N mutant (green) structures correspond to the most represented configurations from the MD simulations obtained by clusterization. Van der Waals contribution between I307 and N307 with I54, R314 and M317 is shown

Site mutation in mutant I307N represents a non-conservative mutation, where a hydrophobic residue is replaced by a polar one, with important neighboring interaction differences associated. In the WT, residue Ile307 is basically surrounded by hydrophobic residues, such as Ile54, Pro304, Phe313 and Met317 as well as by two polar residues Tyr306 and Arg314 distant enough not to sustain hydrogen bond interactions. The introduction of a polar residue in the mutant permits the formation of a hydrogen bond interaction between residue Asn307 and Met317, but in contrast, the hydrophobic interactions with its neighbors worsen. This is reflected in the comparison of the per-residue contribution to the van der Waals interaction energy between the WT and the mutant (Fig. 7). This difference can justify the reduced thermal stability observed in the mutant compared to the WT.

**Fig. 8:**
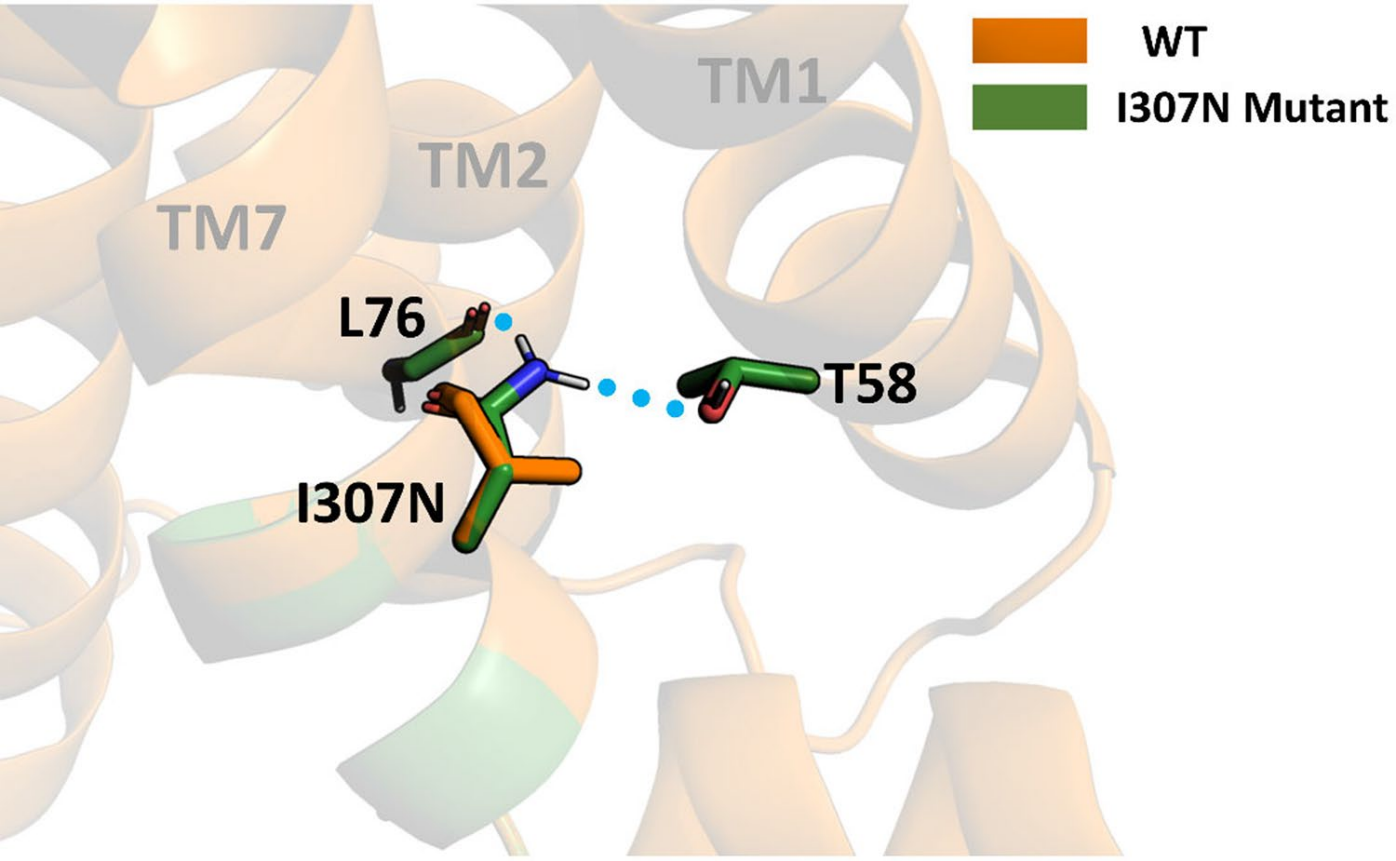
Comparison between WT and I307N mutant on their active states. WT (orange) correspond to the most represented configuration from the MD simulation obtained by clusterization. I307N mutant (green) has been obtained by mutagenesis using PyMol [33]

Inspection of the active and non-active conformations of Rho (5DYS and 1U19) reveals that in the active form TM7 is rotated in such a way that, in the activation process, Ile307 takes the position of Tyr306 of the inactive form. Consequently, in the active form Ile307 is moved into a polar environment close to Thr58 side chain and the backbone atoms of Leu76 (Fig. 8). It is also important to mention that the rotation of TM7 makes Tyr306 to lose a hydrogen bond interaction with Thr58. In contrast, in the active form of the mutant, Asn307 forms two new interactions that are not present in the WT form, adding stability to this conformation, which facilitates the rotation of Tyr306. This difference in interactions could justify that Rho is constitutively activated.

**Fig. 9:**
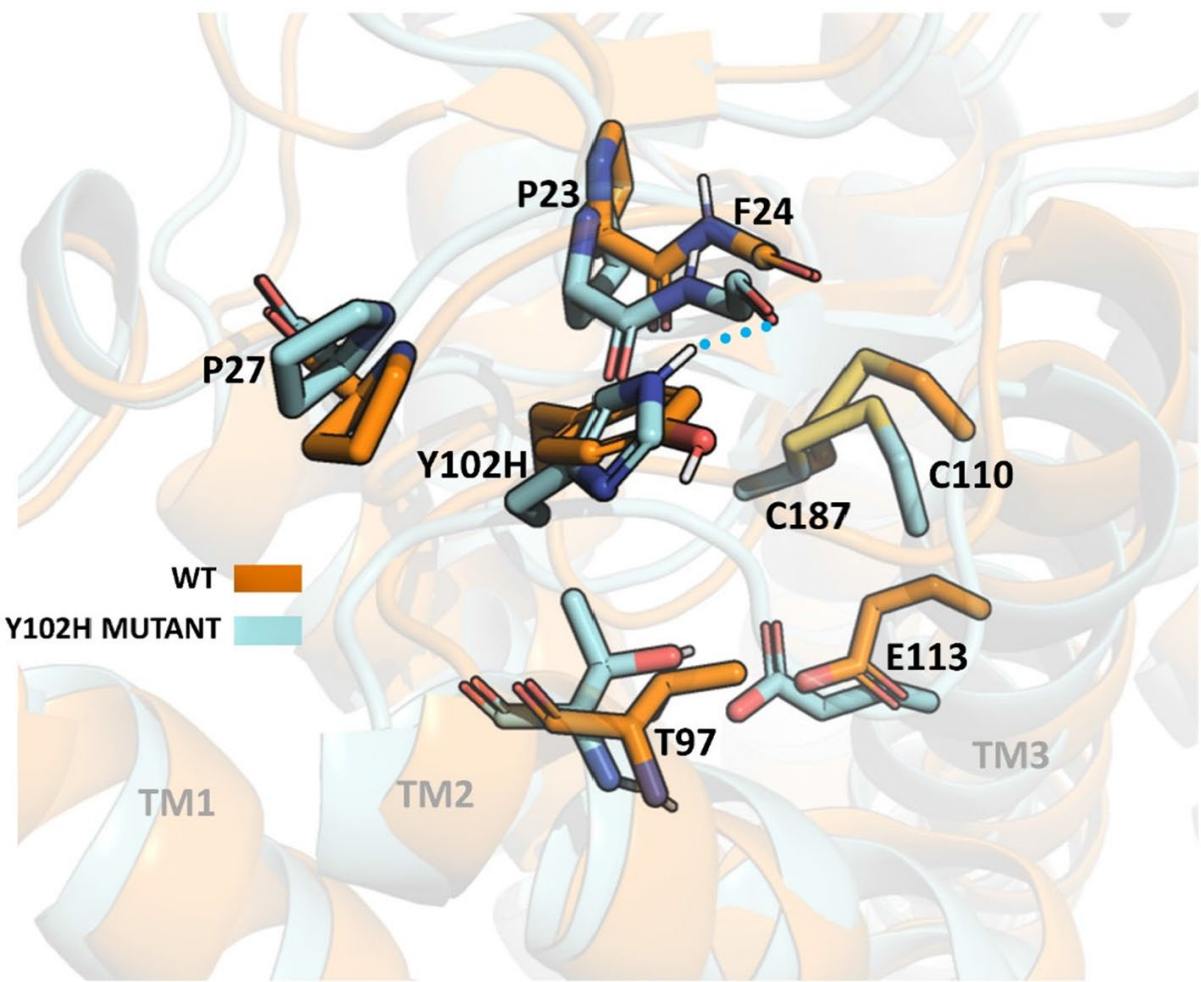
Comparison between WT and Y102H mutant on their inactive states. WT (orange) and Y102H mutant (cyan) structures have been obtained from clusterization and correspond to the most represented ones from the MD simulations

On the other hand, when analyzing the Y102H mutant, no important differences can be observed in structural terms between the mutant and the WT. It has been mentioned that this residue Tyr102 is involved in the maintenance of the proper orientation of the structural core governing Rho stability [34]. Thus, the mutation of the native Tyr residue to His may produce a destabilization of the ECL1 and the N-terminus thereby challenging the binding of retinal. Moreover, since this residue is affecting the N-terminus and protein dynamics, new interactions formed by this His102 (Fig. 9) may be forcing opsin to a particular conformation affecting its stability in the dark inactive conformation, as it has been observed in the thermal stability assay. Thus, it can also be hypothesized that the folding of the protein is affected. It is important to mention that all these neighboring residues that may be affected in both mutants, I307N and Y102H, are conserved in bovine and mice Rho. Thus, the structural effects of these mutations in the bovine and mice backgrounds may be similar.

## Discussion

The retinal degeneration mutants studied here are located in different receptor domains. Gly90 and Ile307 mutations are found in the transmembrane domain, where the helices are closely packed but there is a cavity for retinal binding formed by helices 3, 4, 5, 6 and 7 [35]. In contrast, Tyr102 is located at the extracellular domain in ECL1, which is a compact domain that functions as a “retinal plug”, including two antiparallel β-sheets in the N-terminus and ECL-II. The part of the ECL2 that includes Glu181, penetrates deep into the Rho core, closer to the chromophore [36].

Despite being located at different domains, the G90V and the Y102H mutants presented a similar behavior. Both mutants showed a somewhat lower purified receptor yield and a somehow higher *A*_280nm_/*A*_λmax_ ratio than WT Rho. This may reflect the presence of a small fraction of misfolded protein in the purified sample (Table 1). Likewise, they showed a similar molar extinction coefficient indicating no significant perturbations in the retinal binding pocket in the dark state conformation. Unlike the G90V mutant, Y102H did not present any alteration related to the formation of photointermediates after illumination and this mutant only showed a slight blue shift of 2 nm instead of the 10-nm blue shift observed for the G90V mutant. In the electrophoretic analysis, in both cases, a prominent band appeared below the Rho monomer band. This band has been reported to correspond to an N-terminal truncated product of opsin. These fragments are recognized by the C-terminal antibody used for Western blot, and by having lost the N-terminus, these fragments would not be glycosylated [28, 37].

In addition, both Y102H and G90V mutants showed a great instability in the dark state at high temperature even though the Y102H mutant showed a more compact structure around the Schiff base linkage when compared to the G90V as seen from its hydroxylamine reactivity behavior (Fig. 3). Another of the similarities between these two mutants was a lower chromophore regeneration, which agrees with a decreased protein yield obtained during the purification. It is noteworthy that despite having lower chromophore regeneration than the WT, the Y102H receptor shows similar regeneration initial rate than the WT (Fig. 5C). The same happens for Y102H mutant Gt activation, that has a similar kinetic profile to that of the WT (Fig. 6). However, the functional level of this mutant is decreased and this appears to correlate with the results obtained in the Meta II decay experiment, where G90V mutant showed a slower rate (about double), but the Y102H mutant showed a t_1/2_ more similar to that of the WT (Fig. 5B).

In previous studies with the G90V mutant, this behavior has been attributed to an increase in the required space for the Val side chain, in comparison to Gly, affecting Glu113 [9] amino acid of utmost structural importance in Rho. In the dark state Rho conformation, the chromophore is covalently bound by a protonated Schiff base linkage to Lys296 at the 7TM helix. This positive charge is stabilized by an electrostatic interaction with the Glu113 carboxylate side chain that serves as a counterion [38]. If Glu113 is affected, it is likely that the chromophore orientation may be also altered by Val due to the fact that the chromophore is oriented almost parallel to TM 3 involving amino acids 113, 114, 117, 118, and 120. Moreover, the hydrophobic chain in G90V would either not allow a water molecule in the vicinity of Glu113 and the Schiff base to be accommodated or decrease the water molecule affinity [11]. This water molecule plays an important role in the deprotonation step of the Schiff base in the Meta II state [39].

In the case of the Y102H mutant, Tyr102 is a conserved amino acid in the GPCR Rho subfamily [40], and naturally occurring mutations at this position have not still been reported in humans but only in mice that were generated to study RP disease [13]. The observed effects caused by this mutation might be related to the fact that Tyr102 is part of the structural core governing Rho stability. This core includes several clusters, the largest one, in which Tyr102 is found, and surrounds the conserved disulfide bond between Cys110 and Cys187 residues lining the retinal binding pocket [41]. Here, 90% of these amino acids were predicted to be part of the core causing misfolding upon mutation [42].

The largest core consists of parts of TM helices III–V, ECL1 and ECL2. Specifically, it includes residues 9, 10, 22–27, 102–116, 166–171, 175–180, 185–188, 203–207 and 211. Cys110 and 187 form the disulfide bond and are also part of this core. A great portion of this region overlaps with the 11CR binding region which could be related to the low chromophore regeneration of this mutant. Taking into account the presumed strategic location of amino acid 102, mutation of the native Tyr residue to His allows formation of a hydrogen bond with Phe24 (Fig. 9) at the N-terminus of the protein. This may affect the specific interactions at the ECL1 and N-terminus and may be forcing opsin to adopt a particular conformation that would affect the thermal stability of the receptor as seen in the thermal stability experiment. Interestingly, Pro23 (site of the archetypical misfolding RP mutation P23H) is found at the N-terminus of Rho very close to the Y102H mutation site. These observations highlight the relevance of a precise structural arrangement of the extracellular domain of Rho that is critical for optimal folding of the receptor.

Ile307 is found at a distal region from the other two mutations, at the cytoplasmic domain, and its thermal stability was not severely reduced as in the case of the other two mutations, accounting for only 80% decrease compared to WT stability (Fig. 4). The high percentage and rate of chromophore regeneration together with a slower retinal release in this mutant could be related to the uptake and release of retinal. According to the Meta II crystal structure, retinal must go through a complex elongation and torsional motions of its polyene chain and of the β-ionone ring during its binding process [43]. Moreover, the reorganized TM7 bundle displays not only the cytoplasmic crevice as a binding site for α-Gt subunit but also two opening sites into the hydrophobic membrane layer named opening A, located between TM1 and TM7, and opening B, between TM5 and TM6 [43–45]. A continuous retinal channel through the protein which connects these two nonpolar openings was identified by means of computational studies. There, the 11CR would be uptaken through opening A and all-*trans*-retinal released through opening B [46]. It is possible that the substitution of Ile for Asn at residue 307 in TM7, which would lie close to opening A, may improve the entry of 11CR through the channel. However, although studies have been carried out on the effect of channel mutation on uptake and release of the retinal ligand, they did not conclusively prove the actual existence of such a channel [47]. Moreover, modeling studies on all-*trans*-retinal release from Rho suggested that retinal could enter the binding pocket without passing through the membrane [48].

Regarding the low Gt activation and the altered kinetics shown by the I307N mutant, recent studies have found that Ile307 and Tyr306 residues play a key role in the Rho activation pathway [49]. Upon Rho activation, when retinal isomerizes from 11-*cis* to all-*trans*, due to photon absorption, the β-ionone ring moves up vacating the cavity in the inactive state between TM3, TM5 and TM6 [50]. The cytoplasmic side of TM6 moves away from the rest of the TM bundle exposing several residues initially inaccessible. Many of these residues participate in triggering the mechanism for GDP release in G proteins [51]. In the WT, residue Ile307 is mainly surrounded by hydrophobic residues, such as Ile54, Pro304, Phe313 and Met317 as well as by two polar residues Tyr306 and Arg314 distant enough to not sustain hydrogen bond interactions. In the mutant, polar interactions of residue Asn307 do not improve much except for a novel hydrogen bond interaction with Met317. In contrast, interactions with its hydrophobic neighbors worsens. This is reflected by the residue contribution to the van der Waals interaction energy in the WT and the mutant (Fig. 8). This difference can justify the reduced thermal stability of the mutant compared to the WT in spite of the distant location of this position with regard to the retinal binding pocket.

Inspection of the active and non-active conformations Rho reveals that in the active form TM7 is rotated in such a way that Ile307 takes the position of Tyr306 in the inactive form. Consequently, Ile307 is now in a polar environment close to Thr58 side chain and the backbone atoms of Leu76 (Fig. 9). It is also important to mention that the rotation of TM7 makes Tyr306 to lose its interaction with Thr58 via a hydrogen bond. In contrast, in the mutant Asn307 forms two new interactions that are not present in the WT form, adding stability to this active conformation, facilitating the rotation of Tyr306. This could explain the constitutive activity of the I307N mutant [13] and its unusual kinetic profile.

Finally, it should be noted that the Y102H and I307N mutations have not been found in humans to date, and therefore, their physiological relevance may be discussed. However, it is worth highlighting that these mutant systems are the subject of recent interest, and particularly the I307N mutant mouse has become increasingly used to help unraveling the missing details of the molecular mechanism of retinal degeneration associated with retinitis pigmentosa [52–54].

## Conclusion

The Y102H and I307N Rho mutations causing retinal degeneration in mice appear to shift the inactive–active equilibrium of the photoreceptor protein towards its active conformation. The I307N mutant shows an altered functional profile which may be associated with structural changes from the top of helix 7 to the C-terminus of Rho. In the case of the Y102H mutant, this mutation may affect retinal binding, at the extracellular side of the receptor which represents a compact three-dimensional structure essential for proper conformational folding of the protein. A detailed knowledge of the structural and functional alterations caused by retinal degeneration mutations is needed for the devise of effective treatment such as those based on the use of small ligands that can reverse the deleterious effects of the mutations.

## Supplementary Information

Below is the link to the electronic supplementary material.

Supplementary file1 (PDF 398 kb)

## Data Availability

The data sets generated during and/or analysed during the current study are available from the corresponding author on reasonable request.
