# Supplementary material for: New insights into the molecular mechanism of rhodopsin retinitis pigmentosa from the biochemical and functional characterization of G90V, Y102H and I307N mutations
Source: Cell Mol Life Sci. 2022 Jan 7;79(1):58. doi: 10.1007/s00018-021-04086-0 (PMC8741697; doi:10.1007/s00018-021-04086-0)
Supplement: Supplementary file 1 — Supplementary file1 (PDF 398 kb) [file 18_2021_4086_MOESM1_ESM.pdf]

Supplementary Material

New insights into the molecular mechanism of rhodopsin retinitis pigmentosa from the biochemical and functional characterization of Y102H and I307N mutations

María Guadalupe Herrera-Hernández, Neda Razzaghi, Pol Fernandez-Gonzalez, Laia Bosch-Presegué, Guillem Vila-Julià, Juan Jesús Pérez, Pere Garriga

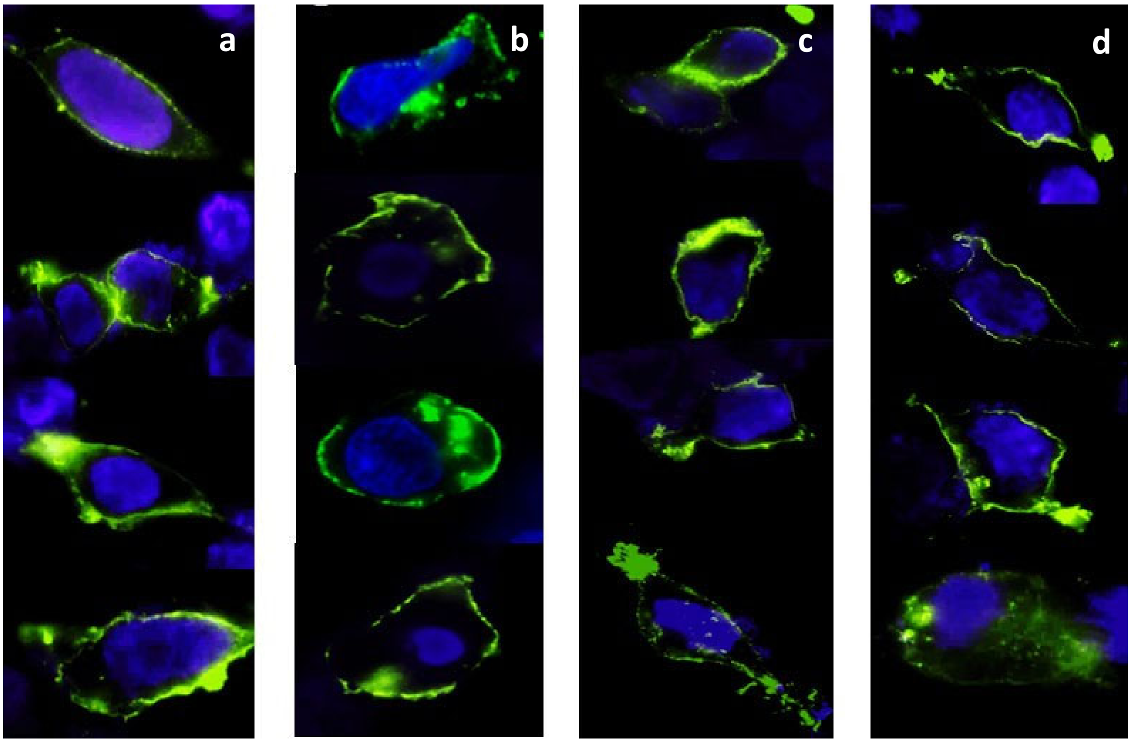

Fig. S1 Subcellular localization of WT and mutants expressed in HEK 293S GnTI^{-} cells. **a)** WT Rho, **b)** G90V mutant, **c)** Y102H mutant, **d)** I307N mutant. Cells were immunolocalized 24 h after transfection

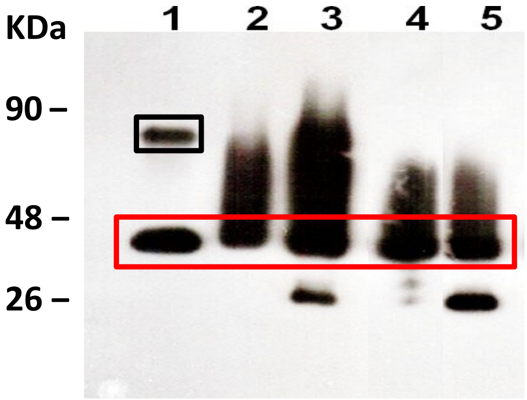

Fig. S2 Western blot analysis of the immunopurified WT and RP mutants. 1: Rho, 2: WT, 3: G90V, 4:I307N and 5: Y102H. Rho monomeric band is enclosed in a red box (~40kDa). The Rho dimeric band, corresponding to the protein obtained from ROS, is enclosed in a black box (~75kDa).

Table S1 Initial rates derived from the plots depicted in Fig 3 corresponding to the chemical stability assay. The relative values of the mutants, with regard to that of WT Rho taken as 1, are also indicated.

	Initial rate (min^{-1})	Relative values
**WT**	0.00118 ± 0.0004	1
**G90V**	0.04548 ± 0.009	38.5
**Y102H**	0.00530 ± 0.003	4.5
**I307N**	0.00219 ± 0.002	1.8
